# Comparative Skeletal Muscle Proteomics Using Two-Dimensional Gel Electrophoresis

**DOI:** 10.3390/proteomes4030027

**Published:** 2016-09-09

**Authors:** Sandra Murphy, Paul Dowling, Kay Ohlendieck

**Affiliations:** Department of Biology, Maynooth University, National University of Ireland, Maynooth, Co. Kildare, Ireland; sandra.murphy@nuim.ie (S.M.); paul.dowling@nuim.ie (P.D.)

**Keywords:** difference in-gel electrophoresis, isoelectric focusing, mass spectrometry, muscle fiber type, muscle plasticity, muscle proteomics, muscular atrophy, polyacrylamide gel electrophoresis, protein separation, skeletal muscle

## Abstract

The pioneering work by Patrick H. O’Farrell established two-dimensional gel electrophoresis as one of the most important high-resolution protein separation techniques of modern biochemistry (*Journal of Biological Chemistry* **1975**, *250*, 4007–4021). The application of two-dimensional gel electrophoresis has played a key role in the systematic identification and detailed characterization of the protein constituents of skeletal muscles. Protein changes during myogenesis, muscle maturation, fibre type specification, physiological muscle adaptations and natural muscle aging were studied in depth by the original O’Farrell method or slightly modified gel electrophoretic techniques. Over the last 40 years, the combined usage of isoelectric focusing in the first dimension and sodium dodecyl sulfate polyacrylamide slab gel electrophoresis in the second dimension has been successfully employed in several hundred published studies on gel-based skeletal muscle biochemistry. This review focuses on normal and physiologically challenged skeletal muscle tissues and outlines key findings from mass spectrometry-based muscle proteomics, which was instrumental in the identification of several thousand individual protein isoforms following gel electrophoretic separation. These muscle-associated protein species belong to the diverse group of regulatory and contractile proteins of the acto-myosin apparatus that forms the sarcomere, cytoskeletal proteins, metabolic enzymes and transporters, signaling proteins, ion-handling proteins, molecular chaperones and extracellular matrix proteins.

## 1. Introduction

Efficient protein separation is a prerequisite for a variety of bioanalytical applications [1]. Physicochemical parameters such as size, charge and solubility of individual polypeptides have been extensively exploited to develop sophisticated techniques for the isolation of specific protein species. Electro-focusing methods and one-dimensional gel electrophoresis (GE) are long established methods of protein biochemistry. Conventional isoelectric focusing (IEF) separates proteins by differences in their isoelectric point (p*I*) whereby a pH gradient along the length of a gel provides the support system for protein migration until they reach their p*I*-value with no net charge [2]. In contrast, one-dimensional gel electrophoresis in the presence of an anionic detergent, such as sodium dodecyl sulfate (SDS), is based on the separation of denatured and structurally linearized polypeptides within complex protein mixtures. Electrophoretic mobility patterns mostly depend on size differences of the denatured molecules due to the introduction of an overall negative charge [3,4,5]. However, since many protein species exhibit a similar net charge or relative molecular mass, one-dimensional gel bands are often heterogeneous in composition. Once it became clear that biochemical techniques focusing on only one parameter have relatively limited separation capacity, alternative approaches were attempted. Especially the sequential usage of two independent methods promised the separation of proteins at higher resolution. The technical realization of this ground-breaking concept was the beginning of a new era in protein biochemistry. The combined property of the p*I*-value of a protein and its molecular size following denaturation was successfully exploited in the development of standardized two-dimensional gel electrophoresis (2D-GE). Historical and technical aspects of combined gel electrophoretic approaches have been extensively reviewed [6,7,8].

Patrick H. O’Farrell’s work set the scene for high-resolution gel electrophoresis [9]. Many other laboratories developed similar approaches or modified the original gel electrophoretic method to adapt this technique to other analytical applications [10,11,12,13]. Both, protein biochemistry and the more recently established field of mass spectrometry (MS)-based proteomics have heavily depended on the 2D-GE method in the past, making this method one of the most commonly employed standard technique of protein separation. In addition, gel-free approaches and the usage of 1D-GE systems are frequently used for the proteomic analysis of complex tissues. The most crucial capabilities of the 2D-GE technique are the simultaneous resolution of thousands of distinct protein species within the same gel system and the reliable determination of their relative molecular mass and p*I*-value, as well as their relative quantity. Especially the application of the 2D-GE method for the efficient separation of different protein isoforms with dynamic post-translational modifications (PTM) has made outstanding contributions in analytical biochemistry. In contrast to other large-scale separation approaches such as liquid chromatography (LC), individual protein spots are visualized in 2D gels so that their status in relation to fragmentation and modification can be directly accessed. Furthermore, gel-separated and -embedded proteins are relatively stable and can be safely stored for long periods of time prior to further analysis [14,15,16,17]. 

This article focuses on the application of the 2D-GE method in basic myology and discusses the enormous scientific impact of this method on recent proteomic studies of normal and physiologically challenged skeletal muscle tissues. This includes the systematic cataloguing of the protein constituents of different contractile fiber types and the findings from surveys of proteome-wide changes during physiological adaptations. Comparative proteomics has also played a key role in the pathobiochemical evaluation of global changes in diseased skeletal muscles [18]. However, this topic is not addressed in this article on the biochemistry of normal, adapting and aging skeletal muscles. Several extensive reviews on the pathoproteomics of neuromuscular disorders have outlined the various proteomic techniques used in the determination of molecular and cellular mechanisms that underlie common muscle diseases [19,20,21,22]. The critical examination of how MS-based proteomics can be used for the systematic identification and biochemical characterization of novel protein biomarkers, which may be exploitable for the future design of improved predictive, diagnostic, prognostic and/or therapy-monitoring assays, has also recently been reviewed [23]. Below sections give an overview of major proteomic studies that have employed 2D-GE methods and analyzed fiber type specification and protein changes during muscle development, fiber type transformation, exercise-induced adaptations, hypoxia-associated alterations, disuse-related muscular atrophy and skeletal muscle aging.

## 2. Two-Dimensional Gel Electrophoresis of Skeletal Muscle Proteins

**Figure 1:**
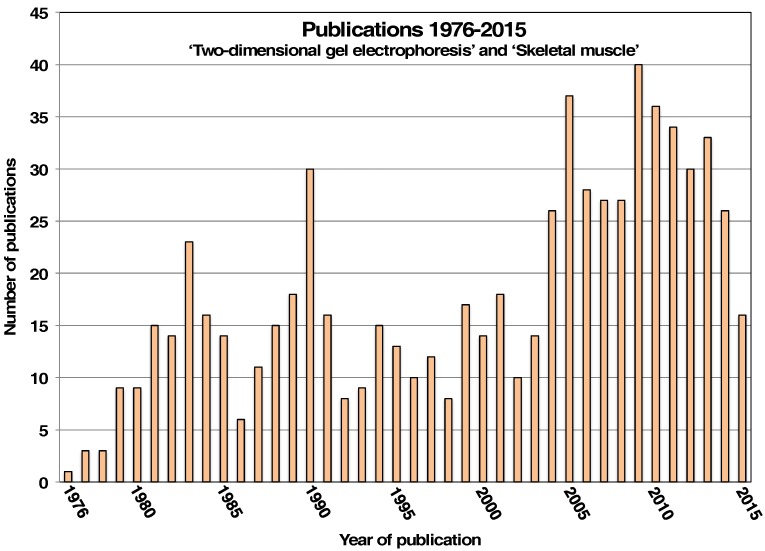
Summary of the number of publication entries with the keywords “two-dimensional gel electrophoresis” and “skeletal muscle” registered with the PubMed databank of the US National Library of Medicine ranging from 1976 to 2015.

The 2D-GE technique is a frequently used and highly reliable bioanalytical method for the systematic assessment of skeletal muscle tissues. Following tissue homogenization under optimized conditions and in the presence of a suitable protease inhibitor cocktail, a large portion of skeletal muscle proteins can be extracted without major complications due to proteolytic degradation and then separated by 2D-GE. The application of the original O’Farrell method [9] or modified and optimized gel electrophoretic techniques [24] played an essential role in the systematic identification and thorough characterization of the protein components that form the functional basis of skeletal muscle contractility. The PubMed databank of the US National Library of Medicine contains nearly 33,000 entries with the keyword “two-dimensional gel electrophoresis” of which over 600 publications are in relation to the combined key words “two-dimensional gel electrophoresis” and “skeletal muscle”, including from the year 2001 onwards nearly 200 papers using MS-based proteomics [25]. Figure 1 summarizes in a histogram the number of published papers per year on this topic since 1976 and shows an increase in the usage of the 2D-GE method after the incorporation of MS-based proteomics for the routine analysis of skeletal muscle tissues since 2004. Over the last 4 decades, the combined usage of IEF in the first dimension and SDS-PAGE in the second dimension has been successfully employed to identify and characterize several thousand muscle-associated or muscle-derived protein species. These muscle protein species belong to the diverse group of regulatory and contractile proteins of the acto-myosin apparatus that forms the sarcomere, cytoskeletal proteins, metabolic enzymes and transporters, signaling proteins, ion-handling proteins, molecular chaperones, extracellular matrix proteins and myokines.

**Figure 2:**
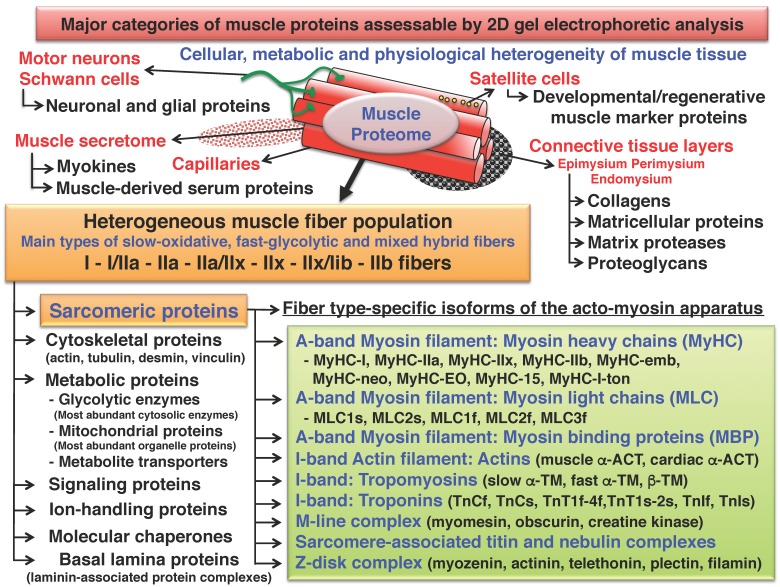
Overview of protein classes from skeletal muscle tissue that can be separated by routine two-dimensional gel electrophoresis using isoelectric focusing in the first dimension and sodium dodecyl sulfate polyacrylamide slab gel electrophoresis in the second dimension. Abbreviations used: ACT, actin; MBP, myosin binding protein; MLC, myosin light chain; MyHC, myosin heavy chain; TM, tropomyosin; Tn, troponin.

As outlined in Figure 2, conventional biochemical approaches and more recently established MS-based proteomic methods have integrated the 2D-IEF/SDS-PAGE technique as a highly suitable method for detailed investigations into skeletal muscle proteins. It is important to mention that skeletal muscle tissues are heterogeneous in their composition and highly dynamic in their response to cellular, metabolic or physiological challenges [26]. The main contractile units of an individual skeletal muscle are presented by diverse fiber populations, consisting usually of slow-oxidative, intermediate fast-glycolytic/oxidative and fast-glycolytic cell types, as well as mixed hybrid fibers. This relates to the histochemically well-defined fiber types I, IIa, IIx and IIb, and the hybrid fibers I/IIa, IIa/IIx and IIx/IIb [27]. The internationally agreed nomenclature of muscle fiber types is based on the distribution of myosin heavy chain (MyHC) isoforms [28]. Besides contractile fibers, muscle tissue contains motor neurons with their extensive myelin sheets, an elaborate network of capillaries, satellite cells and multiple layers of connective tissue, including the epimysium, perimysium and endomysium [29]. Biochemical studies using homogenized tissue preparations have to take into account this cellular heterogeneity of skeletal muscles [21], as well as the presence of actively secreted and passively released fiber-derived proteins that constitute the muscle secretome [30].

Prior to the publication of the original O’Farrell method [9], 2D-GE studies on skeletal muscle employed SDS-PAGE in both dimensions with differing gel concentrations. These early attempts could only separate a few distinct protein spots from muscle ribosomes [31] and the microsomal fraction from skeletal muscles [32]. However, following the publication of high-resolution 2D-IEF/SDS-PAGE methods [9,10,11,12], this new and more refined approach was quickly adapted in the field of basic and applied myology [24]. Initial studies included the analysis of major structural and regulatory proteins of muscle fibers [33,34], the evaluation of human muscle biopsy specimens [35,36] and the identification of contractile protein isoforms in single skeletal muscle fibers [37,38]. In the pre-proteomic era of 2D-GE biochemistry [39], the technique was extensively applied to the detailed analysis of subunit structures and isoform expression patterns of major skeletal muscle proteins and their changes during development, fiber adaptations, contractile fatigue and denervation, as reviewed by Bárány et al. [40]. From 1995 onwards, gel-based surveys and 2D-GE databases became an integral part of skeletal muscle proteomics [41]. Protein changes during myogenesis, muscle maturation, fibre type specification, physiological muscle adaptations, muscle regeneration and natural muscle aging were studied in depth by the original O’Farrell method or slightly modified gel electrophoretic techniques. Below sections review the application of the 2D-IEF/SDS-PAGE method in modern proteomics and its modifications for comparative studies using fluorescent dyes. Included are descriptions of the systematic cataloging of the assessable skeletal muscle proteome, and the comparative proteomic profiling of muscle plasticity in relation to neuromuscular activity versus disuse atrophy.

## 3. Cataloguing of the Skeletal Muscle Proteome Using Two-Dimensional Gel Electrophoresis

MS-based muscle proteomics was instrumental in the identification of several thousand individual protein isoforms following gel electrophoretic separation [21,42,43]. General technical aspects of the most frequently employed 2D-GE methods in the proteomic profiling of crude tissue extracts, subcellular fractions or isolated protein complexes have been extensively discussed and reviewed [15,17,44,45,46]. Over the last few years, several excellent methods books have been published that focus on specific aspects of the many modifications used in routine 2D-GE and proteome analysis protocols. These comprehensive collections of detailed method descriptions have been edited by experts in the field, including Link [47], Reinders and Sickmann [48], Cramer and Westermeier [49], Kurien and Scofield [50], and Marengo and Robotti [51]. In-gel staining methods and routinely used detection technologies for studying 2D protein spot patterns have also been extensively described and critically examined in numerous publications [52,53,54,55,56,57,58,59,60]. The rapidly moving field of MS methodology analyzing peptides obtained from the proteolytic digestion of proteins is the subject of many excellent articles that outline in detail the many instruments and approaches available in modern proteomics research [61,62,63,64]. These optimized gel-based protein separation approaches, highly reliable pre-electrophoretic or in-gel staining techniques and sophisticated MS methods for the unequivocal identification of individual protein species have been extensively used in skeletal muscle proteomics [20,21,22]. In relation to skeletal muscle biochemistry and proteomics, detailed step-by-step descriptions of tissue sample preparation, protein extraction, protein solubilization, IEF, SDS-PAGE, pre-electrophoretic protein labeling, post-electrophoretic protein staining and subsequent MS analysis of proteins of interest following controlled proteolytic degradation have been published in comprehensive methods papers [65,66,67,68]. 

In the first volume of the journal *Proteomics*, launched in 2001, publications by Hochstrasser and colleagues [69] and Dunn and co-workers [70] set the scene for 2D gel-based skeletal muscle proteomics. Their initial studies identified over 70 proteins each from normal mouse and rat skeletal muscle homogenates, including many sarcoplasmic, metabolic and myofibrillary proteins such as various myosin subunits, actin isoforms, regulatory sarcomeric proteins, glycolytic enzymes, mitochondrial proteins and molecular chaperones [69,70]. The application of the 2D-GE method in combination with MS technology for cataloging mouse skeletal muscle was part of extending the SWISS-2DPAGE database to include proteomic maps of major types of tissue [69]. A variety of databases exist for the image comparison of 2D gels, the identification of internet-based gel images, the cataloguing of results from systematic 2D-GE analyses and the integration of electrophoretic and mass spectral data from proteomic analyses, including World-2DPAGE Constellation SWISS-2DPAGE, WEB P.A.G.E, Flicker, GELBANK, Open2Dprot Project, LECB 2-D PAGE Gel Images Data Sets, 2DWG, ProteomeWeb, PHProteomicsDB and pProRep [71,72,73,74,75,76,77,78]. In 2001, the new field of skeletal muscle proteomics was further developed by the systematic identification of sarcoplasmic proteins from several hake species [79] and the mass spectrometric characterization of bovine myosin light chain MLC1f polymorphism following 2D-GE separation [80]. Potential technical shortcomings of 2D-GE for the comprehensive separation of highly complex protein mixtures have often been discussed in the past and compared to the perceived superiority of LC-based methods [17,81,82]. In our opinion, both protein separation techniques should be seen as complementary proteomic methods and be used in combination to achieve the maximum coverage of the assessable proteome of a particular biological specimen.

In the case of skeletal muscle fibers, approximately half of the protein constituents belong to the sarcomere units that are made up of large numbers of isoforms of myosin heavy chains (MyHC), myosin light chains (MLC), actins (ACT), troponins (TN) and tropomyosins (TM). These contractile and regulatory protein species are routinely identified by gel-based proteomics [43], demonstrating the usefulness of 2D-GE for the classification of muscle types and fiber specification. Metabolic enzymes are also highly abundant in muscle tissues and straightforwardly assessable by gel-based proteomics [83]. Prior to outlining the many advantages and bioanalytical applications of 2D-GE in skeletal muscle proteomics in subsequent sections, the below listing highlights certain issues that may hamper gel-based methods and other types of analyses. The most frequently encountered biological and technical complications (and some alternative approaches to avoid these potentially limiting factors) are:

- Possible under-estimation of particular types of proteins, including highly hydrophobic proteins, very high-molecular-mass proteins and low-copy-number proteins. Changing gel conditions, the introduction of suitable pre- and post-fractionation steps, as well as higher sensitivity detection protocols can often overcome some of these technical limitations [84,85,86,87].
- Hypothetical under-representation or 2D streaking of proteins with extreme p*I*-values, which however depends heavily on the particular IEF conditions employed in the first dimensional separation step. Often very acidic protein species form vertical streaking patterns at the pH 3 region and very basic proteins at the pH 11 region. To at least partially overcome this problem, the usage of narrow-range immobilized pH gradients can be applied for zooming in on protein species that do not fall into the commonly applied range of approximately p*I* 3 to 11 [88,89,90,91]. In addition, combining the findings from several different IEF gels in the first dimension with slightly overlapping p*I*-values can be advantageous for producing more comprehensive protein coverage [15,92,93].
- Potentially restricted separation of complex protein mixtures with greatly differing molecular masses using routine 2D-GE approaches. Often the usage of large-scale gels, optimized gradient SDS-PAGE slab gel systems in the second dimension and the reduction of sample complexity can overcome some of these technical problems and be used to cover protein species that do not fall into in the routinely analyzed range of approximately 10 to 250 kDa [67,94].
- Latent cross-contamination of individual 2D protein spots through highly abundant polypeptides that are dragged throughout the 2D gel system due to their exceedingly high density. These abnormal electrophoretic mobility patterns of particular proteins cause a certain degree of 2D streaking, which can be minimized by (i) decreasing the total amount of protein loading; (ii) using very large gel systems with a higher discriminatory capacity and/or (iii) applying optimized pre-fractionation techniques to decisively decrease sample complexity [95,96,97,98]. Artifacts can be kept to a minimum using 50 to 200 μg of total protein in first dimension gels. Lower protein concentrations usually result in weak staining patterns. Comparative studies with fluorescent dyes give optimum results with approximately 50 μg of protein per sample.
- Potential discrepancies between the findings from the densitometric scanning of gel images and the MS-based protein identification in case of a heterogeneous composition of a single 2D protein spot. For example, if a protein spot contains more than one protein species and the most abundant protein is not as susceptible to digestion as the low-copy-number proteins in its vicinity, then the concentration change of this 2D protein spot (as determined by densitometric scanning) may be misleading. However, this analytical complication is a relatively rare occurrence and the use of simple post-fractionation approaches and/or independent verification of gel-based proteomic data by immunoblotting surveys or immunofluorescence microscopical analysis can effectively assess the rate of these kinds of analytical discrepancies [21].

In relation to the analysis of skeletal muscle preparations by 2D-GE, the above listed technical and biological issues may complicate especially the routine analysis of the many very large proteins present in the neuromuscular system, such as titin (3700 kDa), nebulin (800 kDa), obscurin (720 kDa), the ryanodine receptor Ca^2+^-release channel (565 kDa) and dystrophin (427 kDa). However, GE methods can routinely detect fragments of these high-molecular-mass proteins. Many integral or membrane-associated muscle proteins are under-represented in 2D gels and their detailed proteomic analysis has to be carried out with enrichment methods prior to 2D-GE analysis, supplementing LC methods and/or alternative 1D gradient gel systems [99,100,101,102]. The most highly abundant proteins in muscle homogenates are ACTs, MyHCs, MLCs, TMs and TNs. Depending on the overall loading capacity of a particular 2D gel, the high density of some isoforms of these sarcomeric proteins can cause a select amount of cross-contamination in particular regions of a 2D-GE system [67,103].

Despite these bioanalytical limitations, the many technical advantages of the 2D-GE approach far outweigh the potential shortcomings of this extensively used protein separation method, as listed below:

- Extremely reliable protein separation system that can be routinely used in large-scale and high-throughput proteomic surveys. Multi-gel systems using large buffer tanks can run a considerable number of 2D gels in parallel making this approach both cost-effective and highly reproducible for systematic biochemical studies [15,16,17].
- Staining of 2D gels with highly sensitive dyes ranging from colloidal Coomassie Blue to silver stains to a variety of fluorescent dyes can visualize a wide dynamic range of proteins of differing abundance [56,57,58,104].
- Technical provision of a bioanalytical platform that is ideally suited for the subsequent identification of specific protein isoforms and their PTMs [46,47,48]. Many in-gel staining or labeling methods can specifically highlight PTMs, such as enzyme-conjugated lectin labeling or Pro-Q Emerald staining for glycosylation or the fluorescent Pro-Q Diamond dye for phosphorylation [58,105,106,107].
- Direct visualization of proteins of interest as discrete 2D spots, enabling the exact evaluation of the characteristic combination of the p*I*-value and relative molecular mass of a particular protein subunit or isoform. This provides a unique analytical advantage over simpler 1D gel systems that display heterogeneous protein bands or LC methods. Often MS data from LC-based analyses do not given efficient information on sequence coverage to unequivocally determine whether a fully intact protein species or fragments have been detected. In contrast, proteomic data from the analysis of distinct 2D-GE spots can be directly correlated with the electrophoretic mobility and thereby the relative molecular mass of the protein of interest [21].
- Since potential discrepancies between the mass spectrometric identification of a protein and its position in a 2D gel in relation to its p*I*-value and/or molecular mass can be easily assessed, the rate of false positive protein hits can be conveniently measured and swiftly eliminated from the final list of altered protein species. Additional analyses can then determine whether an abnormal or unexpected electrophoretic mobility pattern is due to protein degradation, protein clustering or a technical artifact caused by 2D streaking and cross-contamination [18].
- Rapid and quantitative analyses of paired protein samples can be conducted. An example of an extremely powerful comparative 2D-GE method is the fluorescence 2D-DIGE technique [108] that eliminates gel-to-gel variations by the differential pre-electrophoretic labeling of protein fractions and the subsequent separation on the same 2D gel followed by image analysis [109]. See below section for details on the DIGE method and its application in skeletal muscle proteomics.

Since the beginning of the new millennium, several thousand muscle-associated or muscle-derived protein species and subgroups with particular PTMs have been identified and used to establish the skeletal muscle proteome. A large proportion of the comprehensive cataloguing of total muscle tissue extracts from various species and subtypes of muscle was carried out by 2D-IEF/SDS-PAGE [69,70,110,111,112,113,114,115,116,117,118,119,120,121,122,123,124,125,126]. Subcellular fractions of skeletal muscles have also been studied by 2D-GE and MS analysis [127], including nuclei [128], mitochondria [129,130,131,132], the contractile apparatus [133], cytosol [128] and the muscle secretome [134,135]. In addition, 1D-GE and on-membrane digestion has been used to characterize the sarcolemma [99] and sarcoplasmic reticulum [101] from skeletal muscle preparations. Major classes of PTMs, such as muscle protein nitration, glycosylation and phosphorylation were determined by 2D-GE methodologies [105,106,136,137]. These gel-based proteomic cataloguing studies were supplemented with data from alternative GE methods and a large number of LC-based proteomic investigations [138,139,140,141,142,143,144,145,146,147,148,149,150] to fully comprehend the enormous complexity of the muscle proteome [21]. Details of major 2D gel-based studies for the establishment of the skeletal muscle proteome are listed in Table 1. Within this large cohort of skeletal muscle proteins, fiber type-specific expression patterns of a few hundred muscle proteins have been established by 2D-GE analysis, confirming the molecular and cellular heterogeneity between predominantly fast-twitching and slow-twitching muscles [113,114,115,116,117,118]. This important topic of skeletal muscle physiology and the major changes that occur during fiber transitions is discussed in the below section on comparative proteomics.

## 4. Comparative Skeletal Muscle Proteomics Using Two-Dimensional Gel Electrophoresis

**Figure 3:**
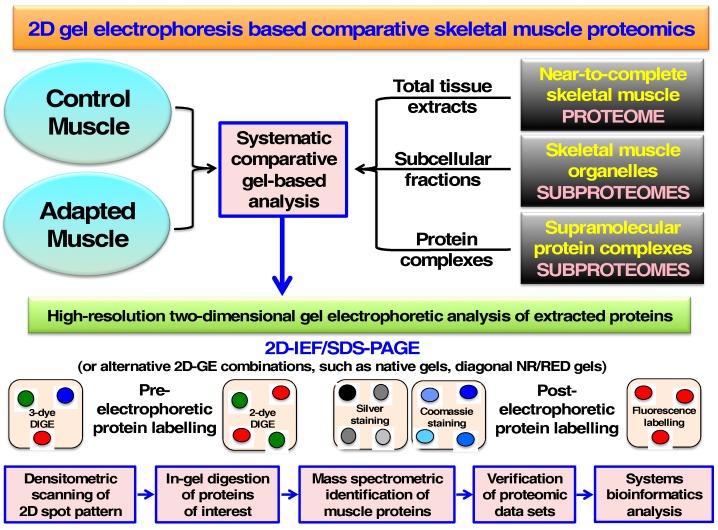
Overview of proteomic approaches routinely used in the profiling of skeletal muscle proteins following separation by two-dimensional gel electrophoresis. Abbreviations used: DIGE, difference in-gel electrophoresis; GE, gel electrophoresis; IEF, isoelectric focusing; NR/RED, non-reducing/reducing; PAGE, polyacrylamide gel electrophoresis.

MS-based muscle proteomics has focused on the systematic determination of protein changes during myogenesis, fiber transformation, exercise-induced adaptations, hypoxia, disuse atrophy and muscle aging. A general proteomic workflow used frequently in comparative skeletal muscle proteomics is outlined in Figure 3.

Comparative proteomic studies have been carried out with (i) total crude tissue extracts representing the assessable and near-to-complete skeletal muscle proteome; (ii) subcellular fractions enriched in specific organelles thereby representing distinct subproteomes; and (iii) isolated supramolecular protein complexes. For the systematic comparative analysis, protein mixtures were separated by high-resolution 2D-GE using usually IEF and SDS-PAGE, but also alternative combinations such as native gels or diagonal non-reducing/reducing gel systems. Protein visualization was performed with both pre-electrophoretic and post-electrophoretic labeling techniques. 2D spot patterns were routinely scanned by densitometry and proteins of interest then identified by in-gel digestion and MS analysis of the resulting peptide populations. The verification of proteomic hits was usually carried out by immunoblot analysis, immunofluorescence microscopy and enzyme assays.

### 4.1. Proteome Signature of Skeletal Muscle Development

Skeletal muscle development is a highly complex cell biological process that is associated with considerable alterations in protein expression patterns. Substantial proteome-wide changes during the phenotypic conversions of myoblasts into post-mitotic myotubes were confirmed by MS studies [151]. Embryonic and adult myogenesis is regulated by a variety of factors, including the transcription factors PAX3 and PAX7 during the initial induction of mesodermal precursor cells and during regenerative processes following muscle injury. Wnt glycoproteins and the myogenic regulatory factors MyoD, Myf5, MRF4 and myogenin play a key role during the segmentation into somites and the formation of the primary myotome [152]. The fusion of myogenic cells results in the formation of multi-nucleated myofibers, which is followed by innervation and muscle maturation. Electro-stimulation of the motor unit system results in the expression of a variety of distinct maturation markers and muscle-specific proteins, including the junctional acetylcholine receptor and the membrane cytoskeletal protein dystrophin. The extremely high level of regenerative capacity of adult muscle fibers is due to the presence of inducible satellite cells, which represent myogenic stem cells that become activated during reactive cycles of fiber regeneration [153]. Skeletal muscle tissue mass is regulated by a complex network of anabolic and catabolic mechanisms and this process includes crosstalk between a variety of regulatory pathways [154]. A key intracellular signaling molecule with differential effects on neuromuscular loading is the transcriptional co-activator PGC-1α (peroxisome proliferator-activated receptor gamma co-activator 1-alpha). Crucial regulatory systems include the TGFβ (transforming growth factor) and myostatin signaling pathway, the NFκb (nuclear factor kappa) and inflammatory cytokines pathway, the IGF1-PI3K-Akt-mTOR (insulin-like growth factor 1-phosphatidylinositol-3-kinase-serine/threonine protein kinase PKB-mammalian target of rapamycin) pathway, the autophagy lysosome, the ubiquitin proteasome, acetylating enzymes and myogenic regulatory factors [155].

The complexity of cell biological mechanism involved in myoblast activation and fiber maturation can be conveniently studied in a systematic fashion by muscle proteomics. Large-scale MS studies of developing muscle fibers promise to establish the regulatory hierarchy of myogenesis and skeletal muscle regeneration. 2D gel-based analyses of skeletal muscle development have involved the proteomic profiling of C2C12 muscle cell culture models with a focus on myoblast differentiation and myotube formation [156,157] and postnatal muscle growth [158,159]. In addition, 1D-GE approaches were used to study changes in the skeletal muscle secretome during myogenesis [160,161,162]. The phenotypic conversion of mono-nucleated myoblasts into differentiated and multi-nucleated myotubes was shown to be associated with considerable changes in highly regulated muscle proteins involved in intercellular signal transduction, intracellular signaling systems, the maintenance of cell shape, the regulation of cell proliferation and apoptosis, as well as protein folding, stabilization and degradation in relation to the cellular stress response [156,157]. Proteomics also confirmed complex changes during the early postnatal period. The developing rat *tibialis anterior* [158] and porcine *longissimus dorsi* muscle [159] showed a time-dependent increase in contractile proteins and drastic alterations in metabolic enzymes, cytoskeletal proteins, molecular chaperones and signal transduction factors. An interesting negative regulator of muscle growth is myostatin, a secreted differentiation factor that belongs to the TGF-β superfamily [163]. *Semitendious* muscles from Belgium Blue bulls that lack myostatin were shown to be characterized by a higher proportion of fast-twitch glycolytic fibers with alterations in contractile protein isoform expression patterns and an increase in myosin binding protein MBP-H [164,165].

### 4.2. Muscle Plasticity and Fiber Type Specification

The physiological regulation of contractile fiber size, fiber type distribution and skeletal muscle mass is closely linked to neuromuscular activity levels. A variety of molecular networks are involved in skeletal muscle plasticity and fibre type specification [155]. Physiological adaptations to changed functional demands and fiber type formation is intimately related to cytosolic Ca^2+^-levels and the activation of the Ca^2+^-calmodulin/calcineurin system. This pathway is involved in the dephosphorylation and subsequent translocation of NFAT (nuclear factor of activated T-cells) into the fibre nucleus, which triggers NFAT-mediated remodeling of muscle gene transcription. Importantly, the activation of the mitogen-activated protein kinase MAPK is a Ca^2+^-dependent process [166]. Thus, Ca^2+^-homeostasis plays not only a central role in the regulation of the excitation-contraction-relaxation cycle and skeletal muscle metabolism, but also influences fiber type specification and muscle adaptations. Adult skeletal muscles are composed of a mixture of slow-twitching fibers, fast-twitching fibers and hybrid fibers. In cell biological terms, the functional and transitional status of an individual skeletal muscle is characterized by the dynamic ratio of slow-to-fast-to-hybrid fibers [167]. The particular mixture of fibers determines the ability of an individual skeletal muscle to generate force. It also influences its susceptibility to contractile fatigue and its properties in relation to shortening kinetics.

Distinct populations of contractile fibers can be distinguished by their cell biological properties (tissue color, cellular diameter, capillary density, mitochondrial content), physiological characteristics (contraction time, power output, relaxation time, relative resistance to fatigue) and biochemical status (aerobic versus anaerobic activities, ratio of oxidative versus glycolytic enzymes, myoglobin levels, triglyceride storage, glycogen levels) [27]. These specific properties of predominantly fast versus slow fibers are reflected by discrete differences in muscle protein density and protein isoform expression patterns. The differential biochemical profile of slow versus fast fibers was confirmed by extensive gel-based proteomic studies that have clearly established several hundred fiber type-specific protein isoforms, including contractile proteins, cytoskeletal proteins, metabolic enzymes, signaling proteins and ion-handling proteins. Major comparative studies of slow versus fast muscles are listed in Table 1 and include the 2D-GE analysis of human *deltoideus* versus *vastus lateralis* muscles [115], pig *longissimus dorsi* versus *soleus* muscles [114], rat *soleus*, *gastrocnemius* and *extensor digitorum longus* muscles [116,118] and mouse *soleus* versus *vastus lateralis* muscles [117], as well as the subcellular study of the nucleus and cytosol from mouse *gastrocnemius* versus *soleus* muscles [128].

Future studies on muscle plasticity can build on these extensive proteomic 2D-GE maps and determine how chronic neuronal, mechanical or metabolic changes may affect the fiber type distribution during physiological muscle conditioning or pathophysiological insults. In general, complex work load-related signaling mechanisms are involved in interrelated pathways with common molecular mediators that induce skeletal muscle hypertrophy versus disuse-related muscular atrophy [154]. The overall process includes neuronal electro-stimulation patterns, mechanical stimulation, stretch-induced fiber alterations, the influence of shear stress and the effects of gravity exposure, as well as hormonal and metabolic factors. The various signals from these varied physiological stimulations present enhanced versus reduced neuromuscular loading and are efficiently transduced into muscle fibers [155]. The cell biological integration alters the response of individual motor units. Thus, neuronal activities, metabolite concentration, oxygen levels, the degree of cellular stress and substrate signaling are key factors that influence changes in the muscle phenotype.

### 4.3. Exercise-Induced Proteome Signature

The effects of enhanced physical activity have been extensively studied in humans and animal models by 2D-GE analysis to determine large-scale adaptations in skeletal muscle and establish time-dependent tissue changes during exercise [168]. Biological issues that have to be taken into account are the intensity of exercise, the training mode, the overall neuromuscular load, the exercise regimen and the specific skeletal muscle types under investigation [169]. Combined transcriptomic, metabolomic and proteomic approaches promise to identify the detailed regulatory mechanisms underlying molecular and cellular changes during and following physical training. Initial proteomic studies suggest that a large variety of protein alterations reinforce the establishment of power performance versus the endurance phenotype [170]. The findings from previous physiological and biochemical studies of exercise-related protein changes were confirmed by proteomics. The exercise-induced proteome signature is influenced by the type and duration of exercise and especially involves the regulation of glycolytic and mitochondrial protein synthesis, and changes in the isoform expression pattern of contractile proteins. Besides the protein systems involved in ATP generation and contractile force, oxygen and metabolite delivery are crucial factors, as well as components that maintain the cellular stress response and anti-oxidant capacity of adapting fibers. Proteomics corroborated the physiological concept that a single bout of intensive exercise affects cytosolic Ca^2+^-release and triggers increased amino acid metabolism. In contrast, chronic endurance training is clearly related to enhanced mitochondrial metabolism and an up-regulation of proteins involved in the citric acid cycle and oxidative phosphorylation [171].

Proteomic profiling of exercise has included the evaluation of protein changes in various human muscles [172,173,174,175,176,177] and established animal models of different training regimes [178,179,180,181,182,183,184,185,186,187,188]. The individual investigations are listed in Table 2. Human exercise studies have focused on (i) the effects of interval training on the *vastus lateralis* muscle [172]; (ii) changes in the mitochondrial proteome from *vastus lateralis* muscle following extended periods of endurance training [173]; (iii) the response of *soleus* and *vastus lateralis* muscles to vibration exercise countermeasures to prevent muscular atrophy in lower limbs due to long-term bed rest [174,175]; (iv) the effects of acute or repeated eccentric exercises on *rectus femoris* muscle [176] and (v) exercise-induced muscle damage and inflammation in *vastus lateralis* muscle following extensive downhill running [177]. Distinct adaptive responses were identified for key muscle proteins, including changes in the ATP synthase and succinate dehydrogenase following interval training [172], a glycolytic-to-oxidative enzyme shift during endurance training [173], altered MyHCs and oxidative enzymes following vibration exercise [174,175], changes in glycolytic enzymes and myosins in response to repeated eccentric exercises [176], and altered expression levels of actin, desmin and calsequestrin in damaged muscles [177].

The proteomic signature of increased neuromuscular loading in animals was evaluated in response to (i) moderate intensity endurance training in rat *plantaris* muscle [178]; (ii) 14 and 60 days of chronic low-frequency stimulation of rabbit *tibialis anterior* muscle [179,180]; (iii) high intensity swimming in rat *gastrocnemius* and *epitrochlearis* muscles [181,182]; (iv) treadmill endurance overtraining in rat *gastrocnemius* muscle [183]; (v) one bout of an exhaustive exercise in rat *gastrocnemius* muscle [184]; (vi) training effects on protein carbonylation in rat *tibialis anterior* and *soleus* muscles [185]; (vii) different stages of endurance training in horse *vastus lateralis* muscle [186]; (viii) endurance training in mouse leg muscles with insulin-like growth factor-mediated gene doping [187]; and (ix) high-capacity versus low-capacity running in rat *soleus* muscle [188]. Endurance training was shown to be clearly related to shifts from glycolytic metabolism to mitochondrial bioenergetics [176,181,182,183,187]. The chronic activation of motor units by electro-stimulation caused a drastic increase of markers of oxidative metabolism and a transformation of contractile proteins to slower isoforms [179,180]. Interestingly, the comparative 2D-GE analysis of *soleus* muscles from rats that were artificially selected as either high- or low-capacity runners identified protein disulfide isomerase PDIA3 as a key component that is associated with the aerobic capacity of slow skeletal muscles [188].

### 4.4. Hypoxia-Related Muscle Adaptations

Although contractile fibers are metabolically robust and remarkably resistant to short periods of oxygen deprivation, very strenuous exercise or physical activity during chronic exposure at altitude cause an adaptive response in the skeletal musculature [189]. A chronic decrease in oxygen levels causes a bioenergetic challenge to metabolically active muscle tissue and is associated with muscular atrophy due to a transient activation of proteolysis and an mTOR-related inhibition of muscle protein synthesis, as well as an overproduction of reactive oxygen species and the stabilization of the oxygen-sensitive hypoxia-inducible factor HIF-1α [190]. Proteomic studies have confirmed hypoxia-induced muscle adaptations using both animal models of oxygen deprivation, i.e., zebrafish in hypoxic tanks [191] and rats in hypoxic chambers [192], and human biopsy specimens from *vastus lateralis* muscle during adaptations to different periods of hypoxia [193,194]. The response to chronic hypoxia is clearly associated with an inhibition of fatty acid oxidation and a decreased expression of proteins involved in the citric acid cycle and oxidative phosphorylation, as well as moderate alterations in the glycolytic pathway [191,192,193,194]. The hypoxia-related loss of muscle mass and decrease in fiber area is probably an adjustment to impaired oxygen diffusion, and the apparent oxidative-to-glycolytic shift in fiber metabolism represents an optimization of bioenergetic processes during exposure to environmental hypoxia.

### 4.5. Proteome-Wide Changes during Disuse Atrophy

A variety of physiological or pathophysiological conditions may lead to muscular atrophy. Prolonged episodes of muscle disuse or neuromuscular unloading are related to traumatic nerve crush, complete denervation, exposure to microgravity, extended bed rest in the severely ill, various forms of immobilization or a general lack of physical activity as for example seen in the elderly. Muscular atrophy has severe effects on the musculature triggering a net loss of skeletal muscle protein mass and contractile strength. In general, muscular atrophy is associated with a drastic decrease in protein synthesis and concomitant increase in the rates of protein breakdown, as well as slow-to-fast transitions in contractile kinetics and an oxidative-to-glycolytic shift in fiber metabolism [195]. A considerable number of gel-based studies have used animal models of denervation, immobilization or extended periods of muscle disuse to determine proteome-wide changes during muscular atrophy [196,197,198,199,200,201,202,203,204,205]. These studies have confirmed the general tendency of atrophying skeletal muscles to undergo a stepwise conversion to faster contractile properties and increased glycolytic metabolism. The proteomic profiling of rat muscles following denervation [196], hindlimb suspension [197] or immobilization by the pin-heel method [198] showed increases in carbonic anhydrase CA3, enolase and fast MLC, as well as decreases in the fatty acid binding protein and slow MLC. As described in the proteomic studies listed in Table 2, neuromuscular unloading is clearly associated with a slow-to-fast transformation process [199,200,201,202,203] and complete denervation triggers extensive isoform switching in slow versus fast isoforms of the contractile proteins MyHC, MLC, TN and TM [204,205]. As already mentioned in above section on exercise proteomics, vibration training can be beneficial to counteract muscular atrophy triggered by extended periods of bed rest [174,175]. The proteomic profiling of disuse atrophy following chronic bed rest has revealed decreases of type I fibers and an increase of hybrid fibers in human *soleus* and *vastus lateralis* muscles. An oxidative-to-glycolytic shift in skeletal muscle metabolism was confirmed by the identification of elevated levels of glycogen phosphorylase and glycolytic enzymes, and a concomitant decrease in mitochondrial enzymes involved in oxidative phosphorylation [175].

### 4.6. Sarcopenia of Old Age

The regenerative capacity of senescent fibers is greatly compromised causing a natural decline in contractile strength during skeletal muscle aging [206]. This functional decline of the neuromuscular system is related to both a gradual loss in skeletal muscle mass and fiber type shifting due to a higher susceptibility of aged type II fibres to muscular atrophy [207]. Sarcopenia of old age has been recognized as a major contributor to frailty in the elderly [208] and affects a large portion of the general population over 75 years of age [209]. The pathophysiological mechanisms of sarcopenia are believed to be highly complex resulting in a multi-factorial etiology. Recent proteomic studies have established extensive changes in the skeletal muscle proteome during the natural aging process [210,211,212], whereby gel-based proteomics has been instrumental in the initial cataloguing of the aged muscle proteome [213,214,215]. Skeletal muscle aging is closely associated with persistent impairments of the peripheral nervous system, which trigger excitation-contraction uncoupling and pathophysiological cycles of denervation and faulty reinnervation. Besides reduced neuronal stimulation, many other factors influence the senescent muscle phenotype, such as chronic inflammation, oxidative stress, hormonal imbalances, lipotoxicity, impaired capillary blood flow, decreased protein synthesis and reduced numbers of inducible satellite cells. Increased stress levels in aged fibres were shown by the proteomic establishment of elevated levels of a variety of molecular chaperones, including the small heat shock proteins cvHsp and αB-crystallin [216,217].

**Figure 4:**
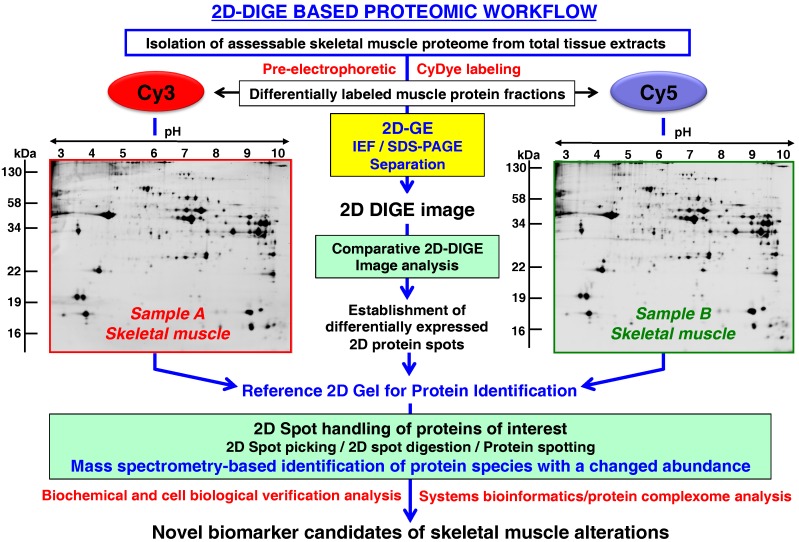
Fluorescence two-dimensional difference in-gel electrophoretic analysis of aging skeletal muscle (Sample A: Young muscle; Sample B: Aged muscle). Abbreviations used: DIGE, difference in-gel electrophoresis; GE, gel electrophoresis; IEF, isoelectric focusing; PAGE, polyacrylamide gel electrophoresis.

A frequently used 2D-GE method in comparative proteomics is represented by fluorescence difference in-gel electrophoresis (DIGE) [108,109,218] and has also been applied to studying sarcopenia of old age [219,220,221,222,223]. The DIGE method was originally described by Minden and colleagues [224] and can be used with differential fluorescent 2-dye or 3-dye systems for minimal or saturation protein labeling prior to 2D-GE [225,226,227]. Figure 4 gives an overview of the routine DIGE analysis of two differing skeletal muscle specimens.

Detailed technical aspects of the DIGE technique for the comparative proteomic analysis of skeletal muscle tissues is outlined in several method papers [65,66,68,228,229]. The development of optimized 2D software analysis tools [230] has boosted the accuracy of quantitative investigations of multiple protein samples on the same 2D gel [231]. The parallel analysis of differentially and pre-electrophoretically labeled proteomes on the same gel greatly reduces gel-to-gel variations, making the DIGE method an excellent comparative tool of analytical protein biochemistry [232,233]. In the field of skeletal muscle proteomics, pre-electrophoretically labeled and 2D-GE separated proteins usually account for a representative proportion of components from the contractile apparatus, structural cytoskeleton, basal lamina, excitation-contraction coupling complex, major metabolic pathways, ion handling systems and regulatory mechanisms [228,234]. For the routine 2D-DIGE analysis of the skeletal muscle proteome, usually 50 μg protein aliquots from individual fractions are differentially labeled with Cy2, Cy3 or Cy5 dyes [65,66,68].

Changes in the abundance of mitochondrial enzymes [128,129,235] and an altered oxidative status of redox-sensitive proteins have been shown to occur in aged muscles [236,237,238]. PTM changes have been reported in phosphoproteins of the contractile apparatus [106,133] and glycoproteins of metabolic pathways [105]. In addition, age-dependent protein nitration and carbonylation appear to play a role in skeletal muscle aging [136,239]. Extensive 2D-GE studies of contractile proteins have confirmed fast-to-slow transitions during both human and animal muscle aging, including a variety of fast and slow MyHC and MLC isoforms [100,133,219,220,221,222,223,240]. These studies demonstrate the bioanalytical robustness and technical suitability of 2D-GE techniques for the efficient separation of the many isoforms of contractile proteins. Proteomic findings support the pathobiochemical concept that age-related fiber transitions towards a slower contractile phenotype are not a primary process, but are based on secondary mechanisms associated with a preferential decline in faster twitching type II fibres [207,214]. This also agrees with the observed glycolytic-to-oxidative shift during muscle aging. Although mitochondrial metabolism is impaired in senescent fibers, the higher susceptibility of glycolytic type II fibers to muscular atrophy appears to cause an overall shift to more oxidative bioenergetics in a slower twitching fiber population from aged skeletal muscles with a drastic decrease in tissue mass [235].

Gel-based meat proteomics has been used to establish reference maps of bovine muscle [113,119], rabbit muscle [122] and pig muscle [114], as well as for studying tissue growth [164,165,241,242] and p*ost mortem* changes in bovine, porcine and chicken muscles [243,244,245,246,247,248]. The findings from meat proteomics are important for maximizing muscle growth, meat production, slaughter methods, muscle-to-meat processing and meat storage [249,250,251].

## 5. Conclusions

Over the last 40 years, protein separation has been carried out by sophisticated 2D-GE techniques and established this method as a highly suitable and versatile approach for the systematic analysis and characterization of the skeletal muscle proteome. In basic and applied myology, the application of the original O’Farrell method or slightly modified versions has resulted in the cataloging of several thousand distinct muscle protein isoforms and the identification of hundreds of fiber type-specific protein species. Gel-based proteomic studies have established a variety of protein changes in physiologically challenged skeletal muscles, including the effects of myogenesis, exercise, regeneration, hypoxia, prolonged disuse and natural aging. In this regard, the 2D-GE method has played an essential role in modern muscle biology and the systematic identification of the molecular components that form the functional units of contractility and adaptability to changed functional demands. The extensive usage of 2D-GE has been instrumental in the establishment of the highly dynamic skeletal muscle proteome signature that is characterized by an extremely diverse population of protein species. Crucial issues in muscle tissue proteomics are optimum protein extraction and efficient protein separation prior to MS analysis. Currently no single protein biochemical method is capable of separating all of the molecular species that constitute the skeletal muscle proteome. The considerable differences in charge, size, solubility and abundance may result in the under-representation of specific subtypes of peptides and proteins. Independent of the specific separation approach, such as gel-based techniques or liquid chromatography, it is currently not possible to cover the entire range of muscle protein isoforms in large-scale and high-throughput analyses. Technical limitations of the 2D-GE method may especially affect the proteomic analysis of very high-molecular-mass proteins, integral membrane proteins and low-abundance proteins. Although liquid chromatography has the advantage of being able to efficiently separate highly hydrophobic proteins, this method cannot routinely determine the characteristic combination of the relative molecular mass and the p*I*-value of a specific protein of interest. Hence, the direct visualization of muscle proteins as discrete 2D spots can be useful to clearly determine whether smaller fragments or a full-length protein have been identified by proteomics. Therefore, using a combination of different biochemical techniques with overlapping separation capabilities for dissimilar subtypes of proteins would be the best way to cover the majority of muscle protein species in comprehensive proteomic studies. With the rapid advances in MS technology, it can be expected that future proteomic investigations will establish an even more refined understanding of the interactions between regulatory proteins, contractile elements, cytoskeletal proteins, extracellular matrix proteins, metabolic enzymes, signaling complexes, ion-handling complexes and molecular chaperones to form the structural and functional basis of the neuromuscular system.

## Figures and Tables

**Table 1:** Major 2D-IEF/SDS-PAGE-based proteomic studies of normal skeletal muscle tissues.

Proteomic Analysis	Tissue and Species	References
Human 2D gel reference maps	Human *vastus lateralis* and laryngeal muscle	Gelfi et al. [111]; Li et al. [112]; Kovalyova et al. [124]
Human fast versus slow muscle fibre type specification	Normal human *deltoideus* and *vastus lateralis* muscles	Capitanio et al. [115]
Mouse 2D gel reference maps	Normal mouse *gastrocnemius* and *quadriceps* muscle	Sanchez et al. [69]; Raddatz et al. [121]
Mouse fast versus slow muscle fibre type specification	Normal and kyphoscoliotic mouse *soleus* and *vastus lateralis* muscles	Le Bihan et al. [117]
Rat 2D gel reference map	Normal rat skeletal muscle from the abdominal wall	Yan et al. [70]
Rat fast versus slow muscle fibre type specification	Normal rat *soleus*, *gastrocnemius* and *extensor digitorum longus* muscles	Okumura et al. [116]; Gelfi et al. [118]
Rabbit 2D gel reference map	Rabbit *gastrocnemius* muscle	Almeida et al. [122]
Bovine 2D gel reference maps	Bovine *semitendinosus* muscle	Bouley et al. [113]; Chaze et al. [119]
Pig fast versus slow muscle fibre type specification	Normal pig *longissimus dorsi* and *soleus* muscles	Kim et al. [114]
Pufferfish and killifish 2D gel reference maps	Skeletal muscles from *Takifugu rubripes* and *Fundulus grandis*	Lu et al. [125]; Abbaraju et al. [126]
Mitochondrial 2D gel maps	Subsarcolemmal and intermyofibrillar mitochondria from various rat muscles	Reifschneider et al. [129]; O’Connell et al. [130]; Lombardi et al. [131]; Ferreira et al. [132]
Contractile apparatus 2D gel map	Enriched acto-myosin apparatus from rat *gastrocnemius* muscle	Gannon et al. [133]
Cytosol and nucleus 2D gel map	Nucleus and cytosolic fraction from mouse *gastrocnemius* and *soleus* muscles	Vitorino et al. [128]
Muscle secretome 2D gel maps	Seretome from cultured muscle cells	Gajendran et al. [134]; Hartwig et al. [135]
2D PTM gel maps of protein glycosylation	Rat leg skeletal muscles	O’Connell et al. [105]; Cieniewski-Bernard et al. [137]
2D PTM gel map of protein phosphorylation	Rat *gastrocnemius* muscle	Gannon et al. [106,133]
2D PTM gel map of protein nitration	Rat leg skeletal muscles	Kanski et al. [136]

**Table 2:** Major 2D-IEF/SDS-PAGE-based proteomic studies of myogenesis, skeletal muscle adaptations, physical activity and muscle aging.

Proteomic Analysis	Skeletal Muscle Tissue	References
Postnatal development	Rat *tibialis anterior* and porcine *longissimus dorsi* muscle	Sun et al. [158]; Xu et al. [159]
Myoblast differentiation and myotube formation	C2C12 cell culture model	Tannu et al. [156]; Casadei et al. [157]
Interval training	Human *vastus lateralis* muscle	Holoway et al. [172]
Endurance training	Human *vastus lateralis* muscle	Egan et al. [173]
Vibration exercise during long-term bed rest	Human *soleus* and *vastus lateralis*	Moriggi et al. [174]; Salanova et al. [175]
Repeated eccentric exercises	Human *rectus femoris* muscle	Hody et al. [176]
Downhill running-induced muscle damage	Human *vastus lateralis* muscle	Malm and Yu [177]
Various types of animal endurance training	Rat *plantaris*, *gastrocnemius*, *tibialis anterior*, *soleus* and *epitrochlearis* muscles; and horse *vastus lateralis* muscle	Burniston [178]; Guelfi et al. [181]; Yamaguchi et al. [182]; Gandra et al. [183]; Magherini et al. [185]; Bouwman et al. [186]
One bout of an exhaustive exercise	Rat *gastrocnemius* muscle	Gandra et al. [184]
Endurance training following gene doping	Various mouse leg muscles	Macedo et al. [187]
Chronic low-frequency electro-stimulation	Rabbit *tibialis anterior* muscle	Donoghue et al. [179,180]
High-capacity versus low-capacity runners	Rat *soleus* muscles	Burniston et al. [188]
Myostatin-related muscle hypertrophy	Belgium Blue bulls *semitendious* muscle lacking myostatin	Bouley et al. [164]; Keady et al. [165]
Hypoxia-induced muscle adaptations	Zebrafish, rat and human *vastus lateralis* muscle	Bosworth et al. [191]; De Palma et al. [192]; Vigano et al. [193]; Levett et al. [194]
Disuse atrophy due to neuromuscular unloading, immobilization or denervation	Rat *soleus*, *tibialis anterior*, laryngeal and *gastrocnemius* muscles	Isfort et al. [196,197,198]; Seo et al. [199]; Moriggi et al. [200]; Ferreira et al. [201]; Basco et al. [202]; Wang et al. [203]; Li et al. [204]; Sato et al. [205]
Skeletal muscle aging	Various aged rat skeletal muscles, including the *gastrocnemius* muscle	O’Connell et al. [105,216]; Gannon et al. [106,133] Kanski et al. [136]; Feng et al. [239]; Doran et al. [217,220]; Piec et al. [240]; Capitanio et al. [221,223]
Sarcopenia of old age	Various aged human skeletal muscles, including the *vastus lateralis* muscle	Gelfi et al. [219]; Staunton et al. [222]

## Abbreviations

The following abbreviations are used in this manuscript:

2D	two-dimensional
ACT	actin
DIGE	difference in-gel electrophoresis
GE	gel electrophoresis
Hsp	heat shock protein
IEF	isoelectric focusing
LC	liquid chromatography
MBP	myosin binding protein
MLC	myosin light chain
MS	mass spectrometry
MyHC	myosin heavy chain
PAGE	polyacrylamide gel electrophoresis
p *I*	isoelectric point
PTM	post-translational modification
SDS	sodium dodecyl sulfate
TM	tropomyosin
TN	troponin
